# Nutrition versus defense: Why *Myzus persicae* (green peach aphid) prefers and performs better on young leaves of cabbage

**DOI:** 10.1371/journal.pone.0196219

**Published:** 2018-04-23

**Authors:** He-He Cao, Zhan-Feng Zhang, Xiao-Feng Wang, Tong-Xian Liu

**Affiliations:** 1 Key Laboratory of Northwest Loess Plateau Crop Pest Management of Ministry of Agriculture, College of Plant Protection, Northwest A&F University, Yangling, Shaanxi, China; 2 College of Horticulture, Northwest A&F University, Yangling, Shaanxi, China; Universita degli Studi della Basilicata, ITALY

## Abstract

Plant leaves of different ages differ in nutrients and toxic metabolites and thus exhibit various resistance levels against insect herbivores. However, little is known about the influence of leaf ontogeny on plant resistance to phloem-feeding insects. In this study, we found that the green peach aphid, *Myzus persicae*, preferred to settle on young cabbage leaves compared with mature or old leaves, although young leaves contained the highest concentration of glucosinolates. Furthermore, aphids feeding on young leaves had higher levels of glucosinolates in their body, but aphids performed better on young leaves in terms of body weight and population growth. Phloem sap of young leaves had higher amino acid:sugar molar ratio than mature leaves, and aphids feeding on young leaves showed two times longer phloem feeding time and five times more honeydew excretion than on other leaves. These results indicate that aphids acquired the highest amount of nutrients and defensive metabolites when feeding on young cabbage leaves that are strong natural plant sinks. Accordingly, we propose that aphids generally prefer to obtain more nutrition rather than avoiding host plant defense, and total amount of nutrition that aphids could obtain is significantly influenced by leaf ontogeny or source-sink status of feeding sites.

## Introduction

The optimal-defense hypothesis suggests that valuable tissues of a plant should be better defended against insect herbivores because young leaves of plants usually have higher growth capacity and are more valuable than older ones [1,2]. Therefore, young leaves generally contain more defensive metabolites than older leaves, and insect herbivores are expected to avoiding feeding young leaves to minimize the plant resistance conferred by defensive metabolites [2]. However, many insects still prefer and grow better on young leaves, which generally contain more nutrients, suggesting that insects may have evolved strategies to minimize the negative impacts imposed by defensive metabolites [1,3]. The relationship between leaf age/ontogeny and plant defense against insects with chewing mouthparts have been examined by some studies, but the influence of leaf age on phloem-feeding insects remains largely unknown [1,2].

Aphids mainly feed on phloem sap of their host plants, which contains a large amount of sucrose and few essential amino acids, providing an unbalanced diet [4]. During their long period of coevolution, aphids have evolved strategies to cope with these constraints by increasing amino acids in the phloem sap and harboring symbiotic bacteria to synthesize essential amino acids [4,5]. Gall-forming aphids can induce strong sinks at the feeding sites, increase the flow of nutrients to the infested tissues and thereby enhance the availability and nutrition quality of the phloem sap [6–8]. In addition, aphids also have evolved behavioral strategies to increase their fitness by choosing host plants and feeding sites within plants [9]. Free-living aphids commonly feed on plant active sinks, like young leaves and reproductive tissues where phloem sap flows to, whereas these plant tissues generally contain more defensive metabolites [10]. Therefore, there seems to be a conflict for aphids between maximizing nutrition and minimizing exposure to toxic metabolites.

Glucosinolates are the major defensive metabolites in Brassica plants, conferring resistance to most of the insect herbivores [11]. Intact glucosinolates are toxic to insects and their toxic effects are enhanced after hydrolysis by the enzyme myrosinase following tissue damage [11]. Since aphids feed on phloem sap using their slender stylets, they cause tiny tissue damage to their host plants and may rarely contact these toxic breakdown products during feeding. However, indole glucosinolates are not stable in aqueous solution and exhibit a strong antifeedant effects on the green peach aphid, *Myzus persicae* Sulzer [12]. However, *M*. *persicae* can grow better on plants containing higher levels of glucosinolates, suggesting that *M*. *persicae* may has, at least partially, adapted to these metabolites [5,13].

For the phloem-feeding insects, the free amino acid in phloem sap is the main nitrogen nutrient, and the amino acid concentration and composition as well as amino acid:sugar molar ratio in phloem sap are important indicators of nutrition quality for phloem-feeding insects [4]. Young leaves generally have higher phloem nutrition quality and more defensive metabolites; however, little is known about the relative importance of these two kinds of metabolites in plant resistance against aphids. In the field, the generalist aphid, *M*. *persicae*, preferred to settle on young leaves of *Brassica oleracea* L. var. *capitata* and *Brassica rapa* [14]. In this study, we first examined the preference and performance of *M*. *persicae* on different-aged leaves of *B*. *oleracea*. Then, we measured the glucosinolates and amino acids in different-aged cabbage leaves. Aphid feeding behavior was monitored and their honeydew excretion rate was quantified. This study intends to assess the relative importance of plant nutrition and defensive metabolites in determining *M*. *persicae* feeding preference and performance on cabbage leaves.

## Results

### *Myzus persicae* prefers and grows better on young cabbage leaves

**Fig 1:**
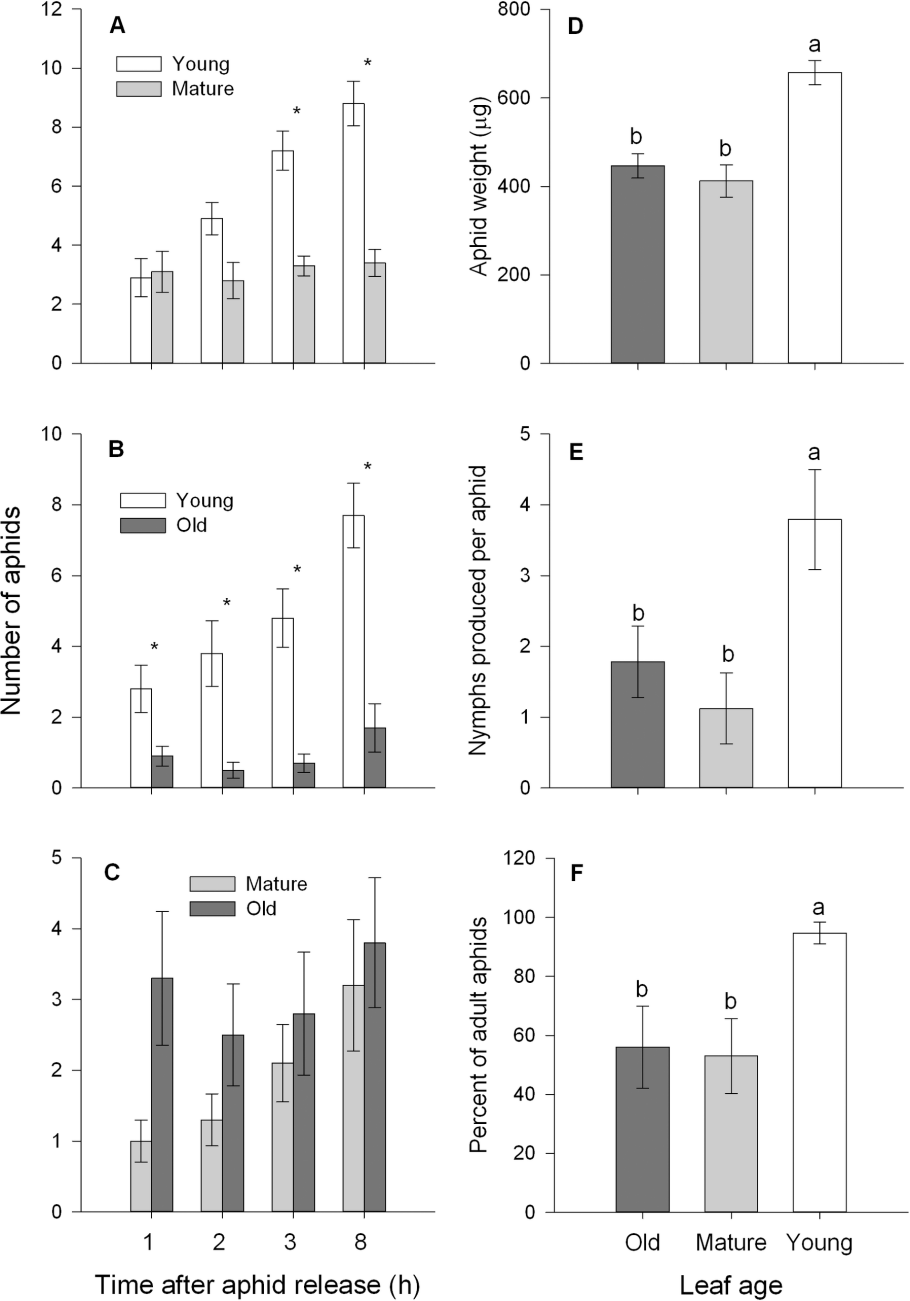
Preference of *M. persicae* for pairs of different-aged leaves (A-C) (paired *t*-test: *P < 0.05); and performance of *M. persicae* in terms of body weight (D), nymph production (E), and number of adults (F). Different letters above the bars indicate significant differences (*P* < 0.05). Values are means ± SE (n = 10).

Significantly more aphids settled on young leaves than on mature leaves after 3 h (*t* = 4.741, df = 9, *P* = 0.001) and 8 h (*t* = 5.063, df = 9, *P* = 0.001; Fig 1A), while more aphids preferred to settle on young leaves compared to old leaves since 1 h after aphid release (*t* = 3.475, df = 9, *P* = 0.007; Fig 1B). By contrast, aphids distributed evenly on mature or old leaves (Fig 1C). Compared with aphids on mature or old leaves, feeding on young leaves resulted in a higher body weight (*F* = 18.534, df = 2, 27, *P* < 0.001; Fig 1D), increased nymph production (*F* = 5.749, df = 2, 27, *P* < 0.01; Fig 1E), and an increased number of adults (*F* = 4.408, df = 2, 27, *P* < 0.05; Fig 1F) at the end of the performance assay.

### Young leaves are more nutritious

**Fig 2:**
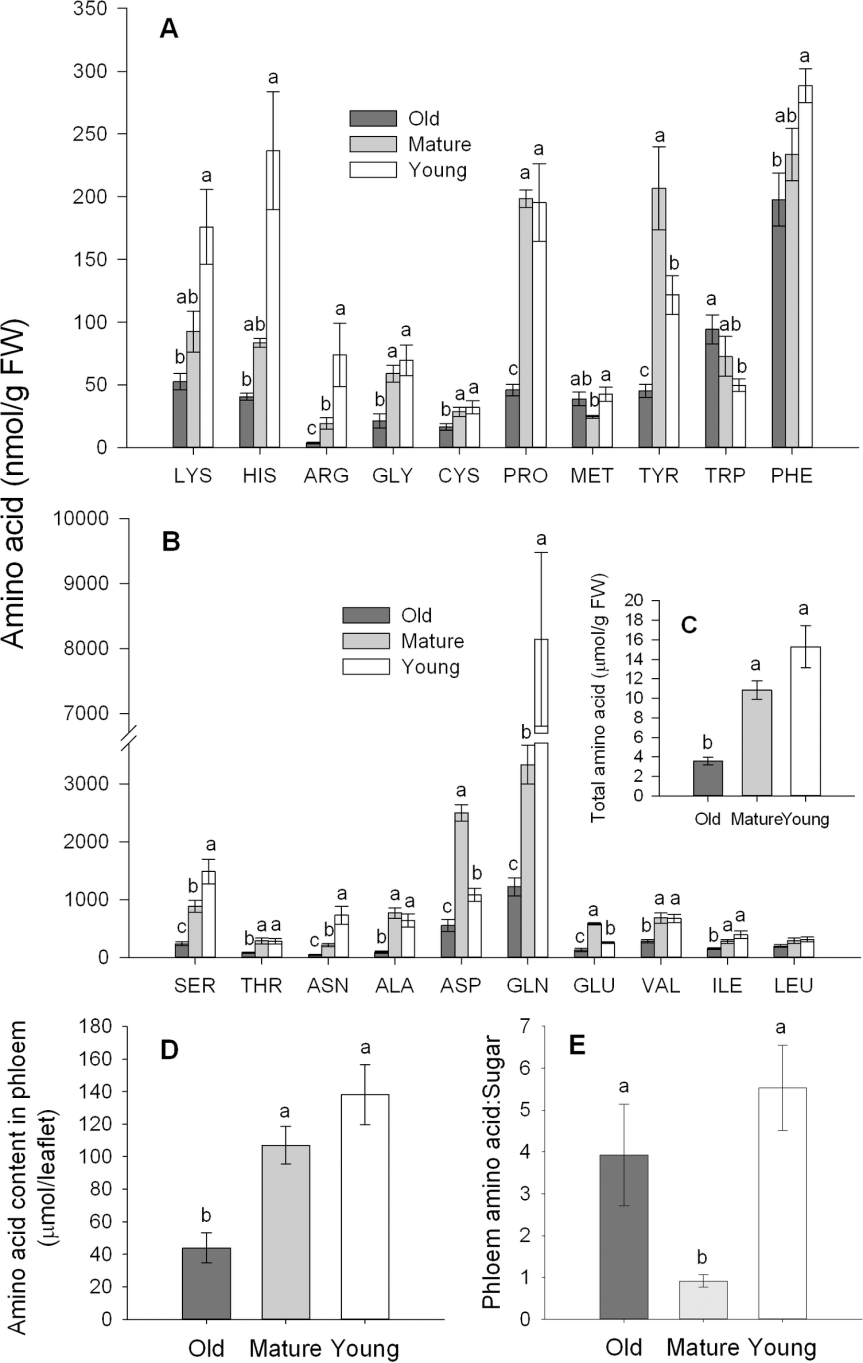
Nutrition quality in cabbage leaves for *Myzus persicae*. Amino acid contents in different-aged leaves (A-C), and the amino acid concentrations (D) and amino acid:sugar molar ratios (E) in the phloem sap. Different letters above the bars indicate significant differences (*P* < 0.05). Values are means ± SE (n = 8).

Young cabbage leaves contained significantly higher levels of arginine (*F* = 17.569, df = 2, 21, *P* < 0.001), serine (*F* = 20.829, df = 2, 21, *P* < 0.001), asparagine (*F* = 30.975, df = 2, 21, *P* < 0.001) and glutamine (*F* = 32.42, df = 2, 21, *P* < 0.001) than old or mature leaves (Fig 2A and 2B). The total amino acid content in the phloem exudate of old leaves was significantly lower than those in mature or young leaves, while total phloem amino acid levels in mature and young were not statistically different (*F* = 12.417, df = 2, 21, *P* < 0.001; Fig 2D). Phloem sap amino acid:sugar molar ratio of young and old leaves were significantly higher than mature leaves (*F* = 15.573, df = 2, 21, *P* < 0.001; Fig 2E).

### Glutamine, methionine and valine stimulate *M*. *persicae* feeding

**Fig 3:**
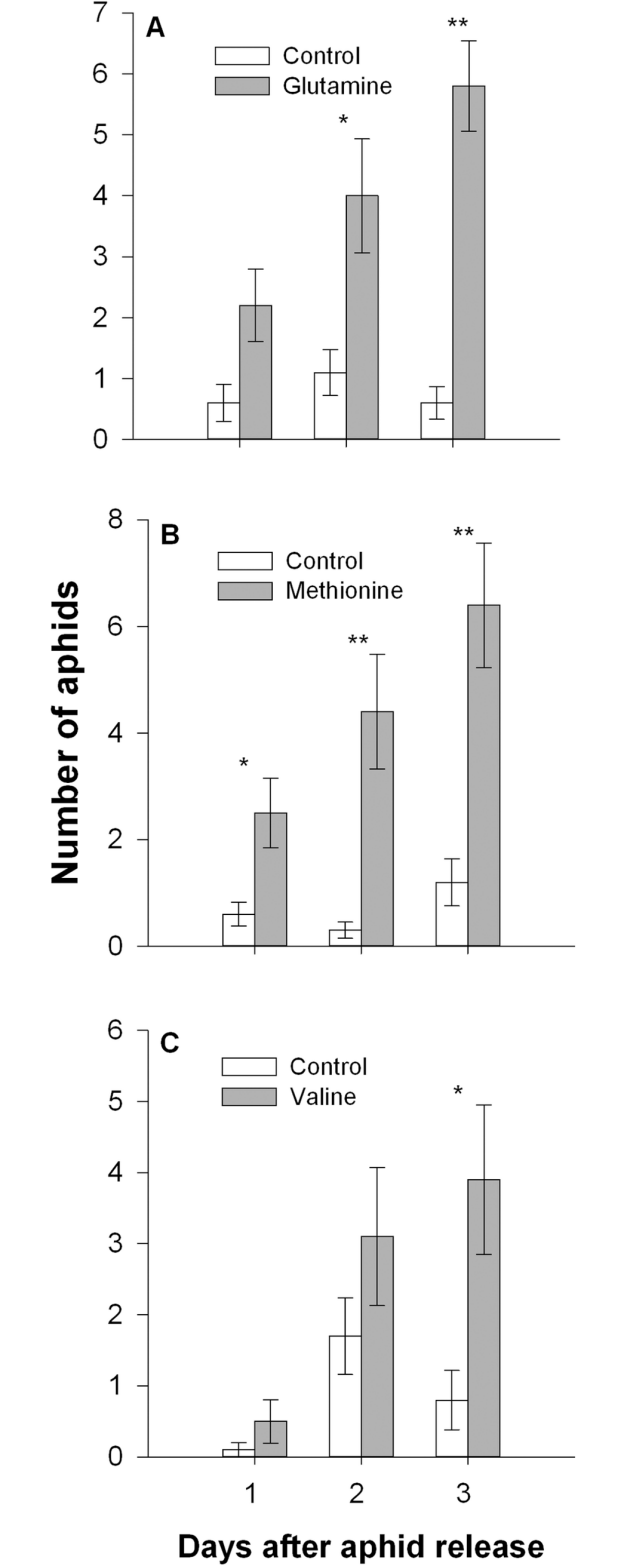
*Myzus persicae* preference for glutamine, methionine, and valine. **Number of adult *M*. *persicae* settled on the 15% sucrose solution (control) or the sucrose solution containing glutamine (A), methionine (B), or valine (C)**. (Paired *t*-test: *P < 0.05, **P < 0.01). Values are means ± SE (n = 10).

Significantly more aphids preferred to settle on the glutamine solution after 2 d (*t* = 2.589, df = 9, *P* < 0.05; Fig 3A) or methionine solution after 1 d (*t* = 2.632, df = 9, *P* < 0.05; Fig 3B) than the control, and the numbers of aphids chose glutamine solution or methionine solution increased with time. After 3 d, aphids also showed a significant preference for the valine solution (*t* = 2.590, df = 9, *P* < 0.05; Fig 3C). There was no significant preference for any of the other amino acid solution.

### Young leaves and aphids that feed on them contain higher glucosinolate concentrations

**Fig 4:**
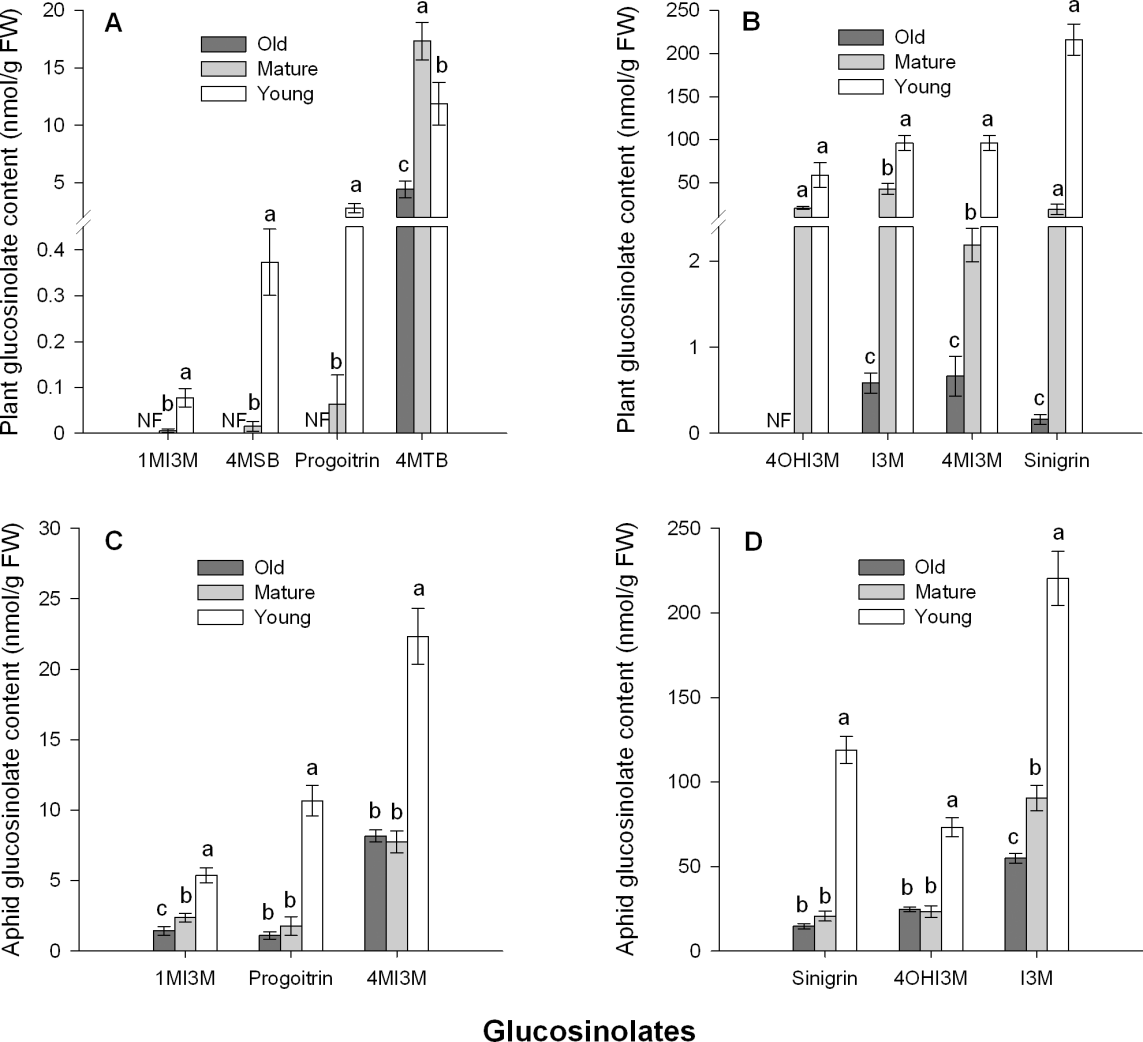
Concentrations of glucosinolates in different-aged cabbage leaves and aphids feeding on them. **Glucosinolates concentrations in different-aged cabbage leaves (A-B), and in *Myzus persicae* feeding on these leaves (C-D).** Different letters above the bars indicate significant differences (*P* < 0.05). Values are means ± SE (n = 8). Glucosinolate side chain abbreviations: 4MTB, 4-methylsulfinylbutyl; I3M, indol-3-ylmethyl; 4MI3M, 4-methoxyindol-3-ylmethyl; 4OHI3M, 4-hydroxyindol-3-ylmethyl; 1MI3M, 1-methoxyindol-3-ylmethyl; 4MSB, 4-Methylsuphinylbutyl.

Young cabbage leaves generally had higher glucosinolate contents than old or mature leaves, while old leaves contained the lowest levels (Fig 4A and 4B). Moreover, aphids that fed on young leaves had the highest glucosinolate levels in their body (Fig 4C and 4D), containing almost two times as much of the indole glucosinolates 1-methoxyindol-3-ylmethyl (1MI3M; *F* = 27.063, df = 2, 21, *P* < 0.001), 4-methoxyindol-3-ylmethyl (4MI3M; *F* = 53.271, df = 2, 21, *P* < 0.001) and indol-3-ylmethyl (I3M; *F* = 102.181, df = 2, 21, *P* < 0.001) than those feeding on old or mature leaves (Fig 4C and 4D).

### *Myzus persicae* has longer phloem feeding time and produce more honeydew on young leaves

**Fig 5:**
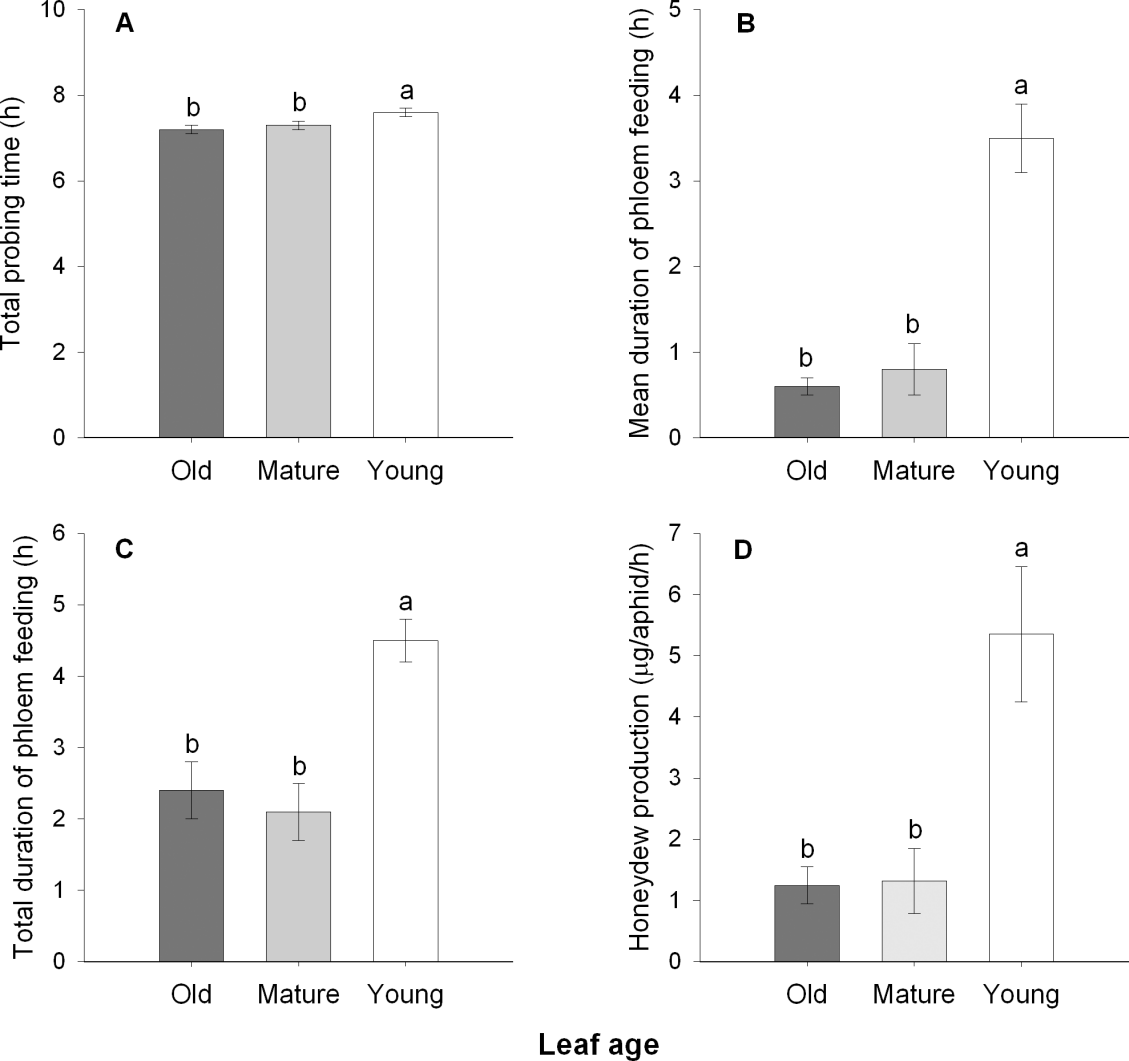
*Myzus persicae* phloem feeding activities and honeydew production rate on different-aged cabbage leaves. Total probing time (A), mean phloem feeding duration (B), total phloem feeding duration (C), and honeydew production (D) by *M*. *persicae* on cabbage leaves. Different letters above the bars indicate significant differences (*P* < 0.05). Values are means ± SE (n = 22–25 for figs A-C; n = 5 for fig D).

Aphids had a significantly longer total probing time when feeding on young leaves than on other leaves (*F* = 4.571, df = 2, 68, *P* < 0.05; Fig 5A). The mean phloem feeding duration of aphids on young leaves was about 4–6 times longer than those on old or mature leaves (*F* = 43.184, df = 2, 68, *P* < 0.001; Fig 5B). Moreover, aphids fed on young leaves had approximately two times longer total phloem feeding time than aphids on mature or old leaves (*F* = 12.638, df = 2, 68, *P* < 0.001; Fig 5C), while honeydew production amount was approximately five times higher than those feeding on other leaves (*F* = 10.407, df = 2, 12, *P* < 0.01; Fig 5D).

### Aphid feeding behavior on cabbage leaves

Aphids feeding on different-aged leaves had similar numbers of probes before their stylets contacting plant phloem (*F* = 0.896, df = 2, 68, *P* = 0.413; Table 1) and spent comparable time on the first probe (*F* = 0.216, df = 2, 68, *P* = 0.806). Aphids had the longest phloem feeding time on young leaves, while they exhibited significantly more numbers of salivation phase (E1) (*F* = 20.914, df = 2, 68, *P* < 0.001) and phloem feeding phase (E2) (*F* = 21.162, df = 2, 68, *P* < 0.001) on old and mature leaves. In addition, *M*. *persicae* infestation did not elicit callose deposits on cabbage leaves (see S1A Fig online), while mechanical wounding resulted in callose deposits (see S1B Fig online).

**Table 1:** Probing behavior of *Myzus persicae* on young, mature, and old cabbage leaves.

EPG Parameters	Young n = 25	Mature n = 24	Old n = 22
Number of probes before 1st phloem phase	7.8 ± 1.7	5.8 ± 1.2	8.5 ± 1.6
Time to 1st probe from start of EPG (min)	3.7 ± 1.8**a**	6.5 ± 1.7**b**	2.5 ± 0.8**ab**
Duration of 1st probe (min)	99.5 ± 31.5	71.5 ± 24.1	34.9 ± 7.3
Time to 1st phloem phase from start of EPG (h)	2.9 ± 0.4	2.3 ± 0.4	2.1 ± 0.4
Number of E1	1.7 ± 0.2**a**	5.0 ± 0.6**b**	6.0 ± 1.0**b**
Number of E2	1.6 ± 0.2**a**	4.8 ± 0.5**b**	5.7 ± 0.9**b**
Mean duration of E1 (min)	0.8 ± 0.1	1.3 ± 0.6	0.8 ± 0.1
Total duration of E1 (min)	1.2 ± 0.2	7.4 ± 4.1	4.7 ± 0.7
Total number of probes	10.1 ± 1.7**a**	17.9 ± 2.0**b**	26.0 ± 3.8**ab**

Data are means ± SE and different letters within each row indicate significant differences (*P* < 0.05). E1, salivation; E2, phloem ingestion; n, number of replicates.

## Discussion

In this study, *M*. *persicae* exhibited a clear preference for young leaves, which agrees with previous findings for other aphid species [10,14,15]. Aphids generally decide to settle on a host plant before their stylets reaching the phloem bundles [9]. We found that *M*. *persicae* could distinguish young leaves from old leaves within one hour, but aphids spent at least two hours reaching the phloem, suggesting that cues that determine aphid preference locate at epidermal or mesophyll cells [9]. Stimulatory and deterrent metabolites as well as plant cell wall composition may influence *M*. *persicae* feeding preference [9,16]. *Myzus persicae* showed a strong preference for the sucrose solution containing glutamine, which is present at a significant higher concentration in young leaves, implying that glutamine may stimulate aphid feeding and contribute to the attractiveness for *M*. *persicae*. Some specialist insect herbivores rely on glucosinolates as settlement cues, which generally were present at higher levels in young leaves [17,18]. However, since indole glucosinolates can act as deterrents to the generalist aphid *M*. *persicae*, this is unlikely to explain aphid preference for young leaves [19]. In addition, plant cells of young leaves are at expanding stage, and thus have flexible cell walls that may increase aphid preference because of lower restriction to aphid stylet penetration and [20]. Although leaf disks were used for aphid choice assay, *M*. *persicae* was also found to prefer to settle on young leaves in the field.

**Fig 6:**
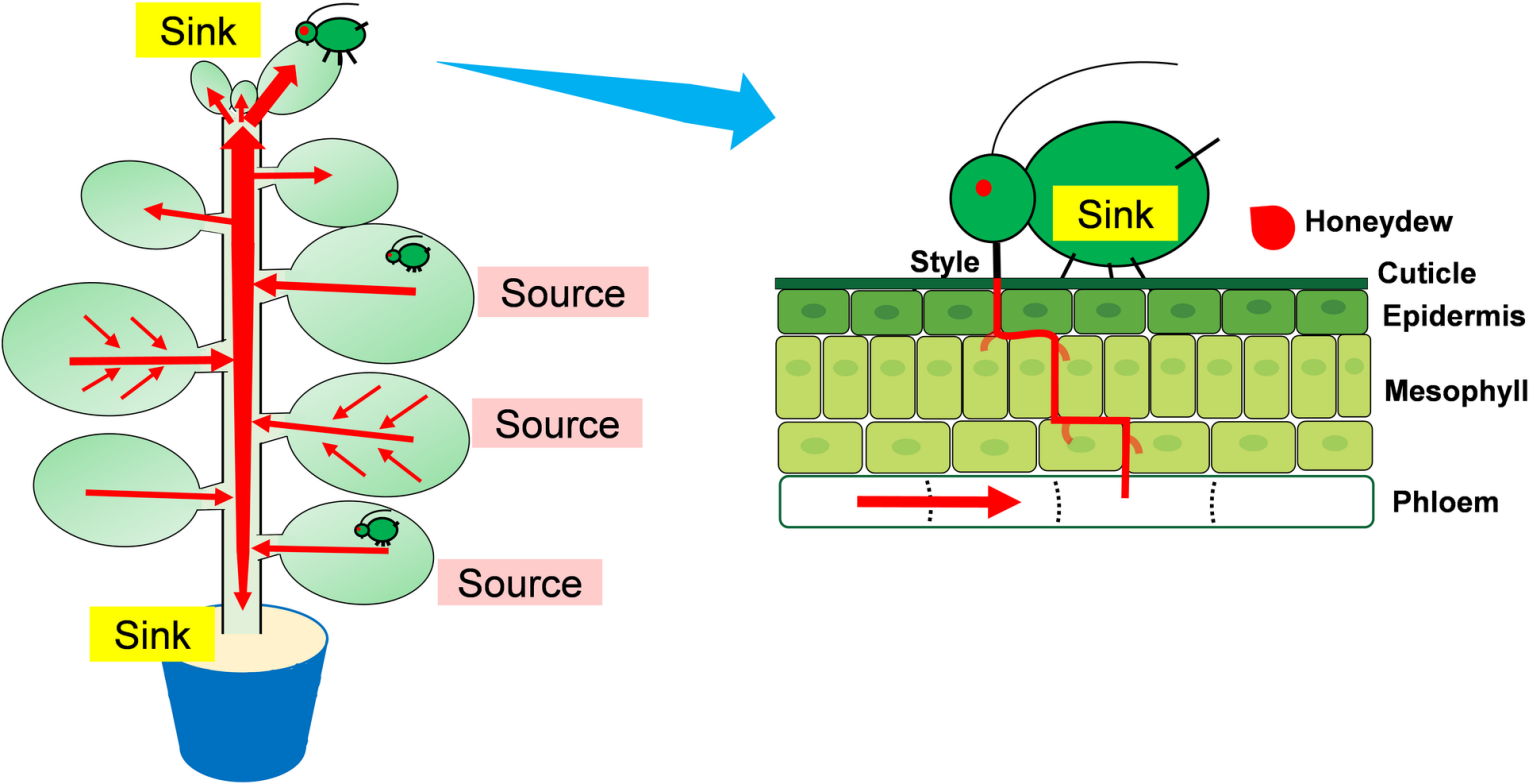
Source-sink status of plant leaves influence host plant resistance to aphids. Young leaves have higher growth capacity than older ones and contain more defensive metabolites. However, aphids can obtain more phloem sap when feeding on young leaves that are natural plant sinks where phloem sap flows to. In contrast, aphids feeding on mature or old leaves compete for phloem sap with natural plant sinks and may thus ingest less phloem sap. To maximize their fitness, aphids generally prefer to obtain more nutrients by feeding on plant sinks rather than avoiding ingesting more plant defense metabolites in these tissues. *Red arrows indicate phloem flow direction*.

Some aphid species can induce galls (induced-sinks) on their host plant, whereas others generally follow the shift of plant meristem and take advantage of natural plant sinks [21]. The EPG data indicated that *M*. *persicae* showed approximately two times longer phloem feeding time (total E2) on young leaves (sink) than on mature or old leaves. Moreover, *M*. *persicae* produced approximately five times as much honeydew per hour when they fed on young leaves, indicating that aphids could obtain more phloem sap by feeding on young leaves. When feeding on mature and old leaves, aphids had to compete for phloem sap with pant sinks, and therefore may ingest fewer phloem sap. The shorter mean phloem feeding time of aphids feeding on mature and old leaves indicated that aphids frequently withdraw their stylets from phloem of these leaves, which is possibly due to the less phloem supply caused by the depletion of phloem sap. Since *M*. *persicae* infestation elicited no visible callose deposits, the reduced phloem feeding time on older leaves is not likely caused by callose block of phloem. Therefore, we propose that the continuous flow of phloem sap from source leaves to young leaves (sink) may support longer phloem feeding time and provide higher amount of phloem sap for aphids than mature and old leaves (Fig 6) [6,22]. Nevertheless, other unknown factors may be also involved in this difference.

Amino acids are the major nutrients for aphids and its composition and concentration significantly influence aphid performance [4]. The amino acid:sugar molar ratio in the phloem sap was about five times higher in young leaves than in mature leaves, indicating a higher nutrition quality in phloem of young leaves. Old leaves showed a similar amino acid:sugar molar ratio in their phloem sap compared with young leaves, but aphids produced five times more honeydew (dry weight) when feeding on young leaves than on old or mature leaves. According to these results (amino acid:sugar molar ratio and honeydew production) and the fact that amino acid and sugar account for more than 90% of the dry material of phloem sap, the total amount of amino acid that aphid ingested from young leaves was approximately 20 times and six times higher than those from mature and old leaves, respectively. And the reduced amino acid ingestion of aphids feeding on mature and old leaves likely account for their lower body weight and fewer nymphs on these leaves [23].

Although previous studies have shown that amino acid concentration in artificial diet or phloem sap is a key limiting factor for aphid performance, there are some exceptions [24–26]. For example, the amino acid transporter mutant (*ant1*) Arabidopsis contained 1.6 times higher amino acid levels in their phloem sap than the wild-type plants, but had no significant influence on reproductive performance of *M*. *persicae* [25]. Moreover, *M*. *persicae* had similar reproduction performance on the amino acid permease mutant (*aap6*) Arabidopsis and the wild-type plants, although *aap6* mutant plants had only one-third amino acid levels in their phloem sap [26]. These results indicate the correlation between amino acid content and aphid performance is more complex than what has been assumed. Aphids feeding on *ant1* or *aap6* consumed comparable phloem sap and therefore total amino acid obtained by aphids between mutant and wild-type Arabidopsis is determined by phloem amino acid concentration, i.e. 1.6 and three times difference [25,26]. This increased or decreased amino acid content obtained by aphids may be below the critical threshold for aphid performance [25,26]. Our results indicated that aphids feeding on young leaves ingested six times more amino acid content than on old leaves and 20 times more than on mature leaves, which may account for the different performance of aphids on these leaves. These results suggest that the total amount of amino acid that aphids obtained play an important role in determining aphid performance, but aphids could adapt to nutrient shortage to some extent.

We found that young cabbage leaves contained the highest levels of glucosinolates, supporting previous findings by Lambdon et al. (2003) [27]. Several studies have shown that glucosinolates are involved in plant resistance to aphids, despite aphids rarely contacting myrosinase during feeding [12,28]. However, in our earlier study, we found that *M*. *persicae*-infested leaves had high contents of indole glucosinolates, but aphids grew better on these leaves [5]. Similarly, the cabbage aphid, *Brevicoryne brassicae* L., also performs better when feeding on reproductive tissues (flowering canopy) than on vegetative tissues (leaves), despite the flowering canopy having higher levels of glucosinolates [15]. In this study, *M*. *persicae* grew better on young leaves that contained higher levels of glucosinolates. These findings suggest that the effects of glucosinolates in plant resistance to aphids has considerable variation, which may be due to aphid adaption or nutrition compensation, and aphids tend to feed on higher nutritious tissues to maximize their fitness, regardless of the higher levels of toxics in these tissues [29,30]. Nevertheless, how *M*. *persicae* adapt to glucosinolates needs to be further studied.

In this study, we found that *M*. *persicae* prefers and performs better in terms of body weight and population growth on young cabbage leaves. However, we cannot exclude a trade-off between productivity and longevity in this research. Aphids feeding on glucosinolate-rich tissues may have reduced longevity, but aphids had shorter generation time and higher fecundity, which may result in more progeny produced earlier in the life-history and over a shorter duration. In addition, nymphs of *M*. *persicae* generally stay where they were born, while adult aphids often move around and choose preferred feeding sites. Thus, it is not natural to confine aphids on specific age leaf for their entire life. Because aphids perform better on sink tissues is a common phenomenon across plant and aphid species, the nutrition other than specific secondary metabolites should be responsible for this phenomenon. We propose that to maximize their fitness, aphids generally prefer to obtain more nutrition rather than avoiding host plant defense, and total amount of nutrition that aphids could ingest is significantly influenced by leaf ontogeny or source-sink status of feeding sites. This hypothesis, however, needs more support by the research in aphid-plant interaction.

## Materials and methods

### Plants and aphids

Cabbage seeds (*B*. *oleracea* L. var. *capitata*, var. “Qingan 70”) were sown in the ground in a greenhouse (23 ± 5°C) under natural light conditions. After two months, similar-sized seedlings were transferred to 10-cm-diameter pots containing soil mixture (peat moss:perlite = 5:1) and placed in a growth chamber under a 14:10 h L/D cycle at 22 ± 2°C and 50% relative humidity (RH). The seedlings were watered with tap water as needed and drenched with water-soluble fertilizer (2 g/L, 20-20-20 + Mg + Trace elements; COMPO Expert GmbH) every five days, receiving five times fertilization in total. The plants were used approximately one month after transplanting, at which time they had about 11–13 leaves. We defined “young leaves” as newly emerged leaves with a diameter smaller than 3 cm, “mature leaves” as fully expanded leaves (about the sixth to seventh emerged leaves), and “old leaves” as those closest to the soil surface. *Myzus persicae* was originally collected from cabbage in the field in Yangling, Shaanxi, China and reared on two-month-old cabbage “Qingan 70” in cages for more than one year in the same growth chamber. No specific permissions were required for collecting aphids in the field in Yangling, Shaanxi, China. Because the field belongs to the Northwest A&F University and our lab rent this field. Thus, we have full right to use it under the law. We confirm that the field studies did not involve endangered or protected species. If not otherwise indicated, adult *M*. *persicae* were used in the following experiments.

### Leaf age and aphid feeding preference

Leaf disks (1 cm diameter) were cut from different-aged cabbage leaves using a steel puncher. Two leaf disks from different age group (i.e., young vs. mature, young vs. old, and mature vs. old) were placed in one Petri dish (9 cm diameter) lined with wet filter paper and approximately 13 apterous adult *M*. *persicae* were introduced to the center of these dishes. The number of aphids can be 10–15, and we found 13 aphids is suitable in our previous study, whereas more adult aphids may lead to crowding. Paired leaves were used because aphids discriminate good or bad host only after penetrating leaf surface using their stylet and may spend more time to find the most attractive leaves (young leaves) when three disks were placed in one petri dish. The number of aphids that had settled on each leaf disk was counted after 1, 2, 3 and 8 h. Ten replicates were performed for each pair of leaf disks.

### Leaf age and aphid performance

This assay was performed with intact plants. Three apterous adult *M*. *persicae* were confined on a young, mature or old leaf using a nylon mesh bag [5]. The petioles were wrapped with cotton to prevent mechanical damage by the bag. After 24 h, the adult *M*. *persicae* were removed, leaving five new-born nymphs on each leaf. After a further 9 d, the aphids were collected and weighed on a microbalance (resolution 0.001 mg; Sartorius MSA 3.6 P-000-DM, Gottingen, Germany), and the numbers of adults and nymphs were counted. Ten replicates were performed for this assay.

### Amino acid preferences of aphids

*Myzus persicae* feeding preference for amino acids was assessed using 15% sucrose solution containing each of the 20 amino acids that make up protein. Thirty-five microliters of 15% sucrose solution (control) and 15% sucrose solution containing 3 mg/mL individual amino acid were confined separately between two layers of stretched Parafilm M on a plastic Petri dish (1 cm high, 3 cm diameter). Then, 12 apterous adult *M*. *persicae* were introduced to each Petri dish and the number of aphids settled on each solution was recorded every 24 hours, lasting for 3 d. Ten replicates were performed for this assay.

### Amino acid and sugar analysis

To investigate the relationship between nutrition quality and leaf age, we measured the amino acid contents in different-aged leaves and their phloem sap. Amino acids in leaves were extracted by grinding the leaves in 0.05 M HCl with a glass mortar and pestle and were analyzed using an LTQ XL linear ion trap mass spectrometer (Thermo Fisher Scientific, Waltham, MA, USA) as described previously [31]. Quantification was achieved by external standard amino acid mixture of known concentrations (AA-S-18, Sigma) supplemented with cysteine, tryptophan, asparagine and glutamine [32]. We used an EDTA-facilitated exudate method to collect phloem sap. The petioles of cut leaves were immersed in 800 μL of 5 mM EDTA solution (pH 7.0) for 3 h in a dark growth chamber (22°C, 100% RH). To determine the amino acid:sugar molar ratio in the phloem, we also determined the concentrations of sucrose, glucose, and fructose in the phloem exudate using the LTQ XL linear ion trap mass spectrometer, as previously described [5]. These sugars are the main carbohydrate in the phloem exudate, whereas all other sugars were less than 1 mg/L and were not calculated. Eight replicates were performed for the analysis of sugar and amino acid.

### Glucosinolates extraction and analysis

To examine the defensive metabolite content in different-aged leaves, we analyzed the concentrations of glucosinolates in young, mature and old cabbage leaves. To inactivate the myrosinase in the leaves, 100 mg of leaves were placed in a 50-mL centrifuge tube and kept in a 96°C water bath for 3 min [33]. The leaves were then ground with a glass mortar and pestle in 1 mL MilliQ water, and the mixture was centrifuged at 12,000 g, 4°C for 15 min. The supernatant was then collected and filtered through 0.22 μm syringe filters. Glucosinolates were analyzed using the LTQ XL linear ion trap mass spectrometer (Thermo Fisher Scientific, Waltham, MA, USA), as described previously [33]. The relative amounts of glucosinolates were calculated according to a standard curve made by 2-propenyl glucosinolate (sinigrin). We collected aphids from different-aged cabbage leaves and analyzed the glucosinolates in aphids as described above. Eight replicates were performed for each treatment.

### Honeydew excretion rate assay

To investigate aphid feeding rate on different-aged leaves, we measured the dry weight of honeydew excreted by aphids feeding on young, mature and old cabbage leaves. Five adult aphids were confined to the abaxial side of each leaf using a clip cage. After 24 h, any nymphs that had been produced were removed and the clip cages were replaced by new clip cages lined with aluminum foil for a further 20 h. These clip cages were placed beneath the aphids so that honeydew that was produced dropped onto the aluminum foil. The aluminum foil was dried to constant weight in a drying oven at 50°C before and after the collection of honeydew and the dry weight of honeydew produced per aphid per hour was calculated. Five replicates were conducted for each treatment.

### Aphid feeding behavior

We monitored the feeding behavior of *M*. *persicae* on young, mature and old leaves using the Giga-8 direct-current electrical penetration graph (DC-EPG) system (W. Fred Tjallingii, Wageningen University, Netherlands). Aphid feeding activities were recorded for 8 h in a Faraday cage at 24°C. An 18 μm diameter gold wire was attached to the dorsum of each aphid using silver conductive glue and then aphids were placed onto leaf surface. Each adult *M*. *persicae* and cabbage plants were used only once. Signal was recorded by the Stylet+d software and the EPG waveforms were recognized and labelled using the Stylet+ software [34]. Both software was provided by Prof. Tjallingii (Wageningen University, Netherlands). EPG parameters were calculated using the Excel workbook for automatic parameter calculation of EPG data 4.3 [35]. We obtained 22–25 successful replicates for each treatment.

### Callose assays

Mixed instar *M*. *persicae* (30 mg) were confined on the abaxial side of leaves using clip cages (1 cm diameter) for 3 d. As a control, some leaves were caged without aphids. Leaves wounded by a needle (0.2 mm diameter) for several times were positive controls. Following collection, the leaves were fixed in ethanol:glacial acetic acid (3:1) and shaken overnight [36]. The leaves were then decolorized in 98% ethanol for 2 h and in 50% ethanol for 2 h, washed 3 times in distilled water, and stained with 0.1% (w/v) aniline blue in 75 mM phosphate buffer (pH 9.5) for 4 h in the dark. Callose deposits were viewed by a fluorescent microscope (EX 330–380 nm; DM 400 nm; BA 420 nm; Nikon eclipse 80i; Nikon Corp., Japan). Three replicates were performed for each group, but only one representative figure was presented.

### Statistical analysis

We found that time had significant influence on aphid settlement on sucrose solution (two-way ANOVA), which may be due to that aphids did not adapt to Petri dish environment and may have difficulties in searching the test solution. Thus, we analyzed the data independently (paired *t*-test), and found that aphids showed clear preference for certain amino acid solution. Aphid preferences for different-aged leaves were analyzed by paired *t*-test. Levene’s and Kolmogorov-Smirnov tests were used to test homogeneity of the variances and normality of the data for aphid weight, EPG results, individual amino acid concentration in the leaves, total amino acid concentration in EDTA exudate, amino acid:sugar ratio in the phloem sap, glucosinolate concentration and honeydew weight. Any data that did not meet these tests were transformed using ln (1 + x). The effects of leaf age on these factors were then analyzed using one-way analysis of variance (ANOVA), followed by Fisher's least significant difference (LSD) tests, at a significance level of *P* < 0.05. All statistical analyses were performed using the IBM SPSS Statistics package 19 (SPSS Inc., Chicago, IL, USA).

## Supporting information

S1 Fig Callose deposits in cabbage leaves in response to *Myzus persicae* feeding and mechanical wounding. (PDF)
