# Supplementary material for: Nutrition versus defense: Why Myzus persicae (green peach aphid) prefers and performs better on young leaves of cabbage
Source: PLoS One. 2018 Apr 23;13(4):e0196219. doi: 10.1371/journal.pone.0196219 (PMC5912751; doi:10.1371/journal.pone.0196219)

Supporting information

Nutrition versus defense: why *Myzus persicae* (green peach aphid) prefers and performs better on young leaves of cabbage

He-He Cao[1,2], Zhan-Feng Zhang[1], Xiao-Feng Wang[2], Tong-Xian Liu[1*]

[1]Key Laboratory of Northwest Loess Plateau Crop Pest Management of Ministry of Agriculture, College of Plant Protection, Northwest A&F University, Yangling, Shaanxi, 712100, China,
[2]College of Horticulture, Northwest A&F University, Yangling, Shaanxi, 712100, China

Short title: Aphids perform better on young cabbage leaves.

***Corresponding author**

E-mail: txliu@nwsuaf.edu.cn (TXL)

S1 Fig. Callose deposits in cabbage leaves in response to *Myzus persicae* feeding and mechanical wounding. (A) Control and aphid infested cabbage leaves. Aphids were confined to leaves by clip cages, and leaves were collected and analyzed after 3 d. Control leaves were treated by empty clip cages. White arrows indicate callose deposits around stoma. (B) Wounding induced callose deposits. Cabbage leaves were wounded by a needle 3 d before sample collection. The bright yellow dots indicated by red arrows are wound-induced callose.

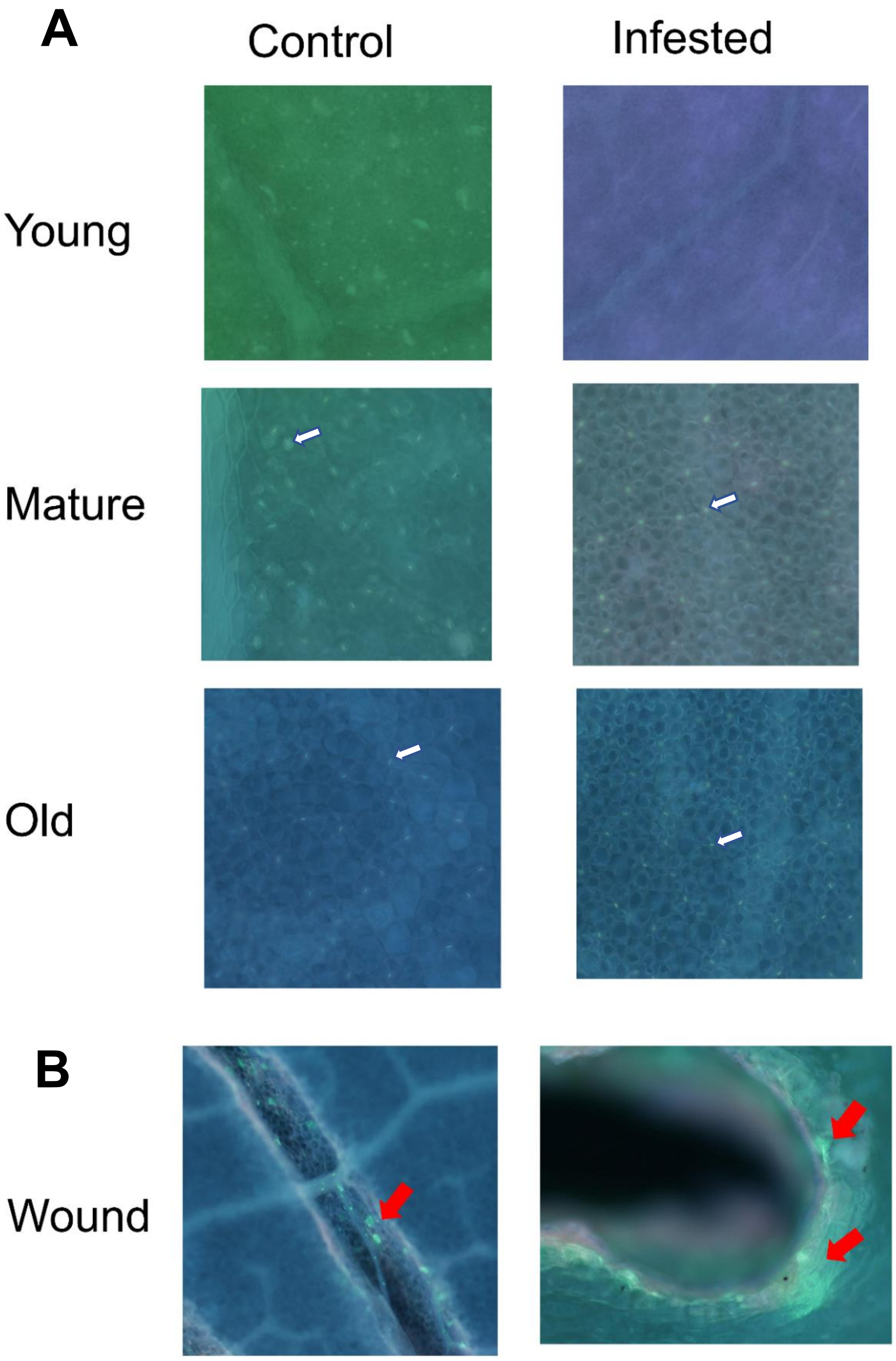

Supplement: S1 Fig — (PDF) [file pone.0196219.s001.pdf]
